# Retrospective analysis of different therapeutic approaches for retroperitoneal duodenal perforations

**DOI:** 10.1038/s41598-022-14278-8

**Published:** 2022-06-17

**Authors:** Can Yurttas, Christian Thiel, Dörte Wichmann, Philipp Horvath, Jens Strohäker, Malte Niklas Bongers, Martin Schenk, Dietmar Stüker, Alfred Königsrainer, Karolin Thiel

**Affiliations:** 1 Department of General, Visceral and Transplant Surgery, University Hospital of Tübingen, Hoppe-Seyler-Str. 3, 72076 Tübingen, Germany; 2 Department for Diagnostic and Interventional Radiology, University Hospital of Tübingen, Hoppe-Seyler-Str. 3, 72076 Tübingen, Germany

**Keywords:** Outcomes research, Prognostic markers

## Abstract

Surgical therapy of duodenal perforation into the retroperitoneum entails high morbidity. Conservative treatment and endoscopic negative pressure therapy have been suggested as promising therapeutic alternatives. We aimed to retrospectively assess outcomes of patients treated for duodenal perforation to the retroperitoneum at our department. A retrospective analysis of all patients that were treated for duodenal perforation to the retroperitoneum at our institution between 2010 and 2021 was conducted. Different therapeutic approaches with associated complications within 30 days, length of in-hospital stay, number of readmissions and necessity of parenteral nutrition were assessed. We included thirteen patients in our final analysis. Six patients underwent surgery, five patients were treated conservatively and two patients received interventional treatment by endoscopic negative pressure therapy. Length of stay was shorter in patients treated conservatively. One patient following conservative and surgical treatment each was readmitted to hospital within 30 days after initial therapy whereas no readmissions after interventional treatment occurred. There was no failure of therapy in patients treated without surgery whereas four (66.7%) of six patients required revision surgery following primary surgical therapy. Conservative and interventional treatment were associated with fewer complications than surgical therapy which involves high morbidity. Conservative and interventional treatment using endoscopic negative pressure therapy in selected patients might constitute appropriate therapeutic alternatives for duodenal perforations to the retroperitoneum.

## Introduction

Duodenal perforation is a rare but life-threatening event. Symptoms of perforation depend mainly on the location. Contrast-enhanced computed tomography (CT) should be the preferred diagnostic modality^1^. In case of open perforation into the abdominal cavity with concomitant peritonitis, patients usually present with a short history of severe abdominal pain and tenderness that may be accompanied by fever and chills. Immediate surgical therapy is considered the treatment of choice. Patients with perforations into the retroperitoneum may describe rather unspecific complaints like epigastric or back pain, nausea and vomiting^2,3^. Comparable to open perforations, perforation into the retroperitoneum with or without abscess formation have mostly been treated surgically^4–10^. However, surgery is challenging and frequently connected with severe complications and mortality rates of up to 30% have been reported in the literature^4,7^. Conservative therapy has therefore been advocated by several authors, reporting on effective treatments in patients with duodenal perforation and stable vital signs but without generalized peritonitis^11,12^. In light of the morbidity entailed with surgery and reports about successful non-surgical treatment approaches for duodenal perforations into the retroperitoneum^7,13,14^, our study aimed to retrospectively assess and compare the outcome of conservative, interventional and surgical treatment of duodenal perforations to the retroperitoneum at our department.

## Patients and methods

### Trial design and data collection

In order to identify patients with perforation of the duodenum into the retroperitoneum, we retrospectively screened all patients diagnosed with international classification of disease (ICD-10) codes K26.0–K26.9 (duodenal ulcer) and K57.02–K57.93 (diverticulitis of the small intestine) that were treated at the Department of General, Visceral and Transplant Surgery, Tübingen, Germany between 2010 and 2021. Patients with open perforation into the abdominal cavity, perforation other than of the duodenum or concomitant circumstances (e.g. acute bleeding) demanding immediate surgical intervention as well as duplicates were excluded from this analysis.

The Ethics Committee at Tübingen University Hospital approved this study and it is registered with project identifier *154/2021BO2*. Informed consent was obtained from all patients and all treatments were carried out in accordance with German guidelines and regulations.

### Diagnostic approach and allocation to therapy

Patients were admitted to our surgical emergency service, transferred to our department from external hospitals or presented to us from other in-hospital services for surgical consultation. Following case history, physical examination and monitoring of vital parameters, establishment of a peripheral venous access and blood tests with blood cultures, all patients received intravenous substitution of crystalloids and, if requested by patients, intravenous analgesia. CT imaging was performed in all patients. Allocation to conservative, interventional or operative therapy followed no predefined criteria, but was made at the consultant’s assessment, unless there were signs of peritonism or sepsis demanding open surgical exploration.

### Conservative therapy

Conservative therapy mainly consisted of intravenous analgesia with non-steroidal anti-inflammatory drugs and, if required, opioid analgesic. Calculated broad-spectrum intravenous antibiotic therapy was initiated early and further on specifically deescalated, if possible, based on antimicrobial testing of microbial cultures. Oral nutrition was temporarily discontinued (nil by mouth) and replaced by either parenteral nutrition or nutrition administered via an endoscopically inserted nasojejunal feeding tube.

### Interventional therapy using endoscopic negative pressure therapy with open pore film drainage

Only endoscopically inserted tubes that were connected with a pump to establish negative pressure therapy were considered as interventional therapy. Endoscopic negative pressure therapy is applied to seal transmural defects in hollow organs and facilitate drainage of wound exudates, debris and liquid duodenal secretions. Therefore the duodenum is actively drained to the intraluminal side. This prevents the digestive secretions from entering the wound with subsequent extraluminal inflammation. The healing process is thereby supported and the internal wound is protected from destructive biliary and pancreatic juices^15^. The technique and underlying principle of negative pressure therapy with open pore film drainage has already been described and published in detail elsewhere^16–21^. Open pore film drains were assembled prior to endoscopic intervention by enveloping a thin open-pore double-layered drainage film (SuprasorbCNP Drainage-Film, Lohmann&Rauscher International, Rengsdorf, Germany) around the tail end of a Redon drain (Medicoplast, Illingen, Germany), gastric tube (Dahlhaus, Petershagen, Germany), or the gastric part of a feeding tube (Freka Trelumina, Fresenius Kabi, Bad Homburg, Germany). The drainage film was then fixed with surgical sutures (Mersilene 1–0, Ethicon, Johnson & Johnson Medical N.V., Belgium). Guided by endoscopy (video gastroscope, Pentax Medical, Tokyo, Japan) and carried with gripping tongs, the drain was positioned intraluminally at the perforation site without usage of an overtube and fixed at the patient’s nose with adhesive tape. Continuous negative pressure of − 125 mmHg was generated with an electronic vacuum device (V.A.C. Ulta; KCI Inc., San Antonio, Texas, USA). Therapy was continued with endoscopic follow-up examinations to exchange the negative pressure device every 3–5 days until full reconstitution of intestinal wall integrity. In case of dislocation, the device was promptly reinserted by endoscopy.

### Open surgery

Open surgery under general anesthesia was performed if deemed necessary by the attending surgeon in charge. A nasogastric tube was inserted during anesthesia and remained at least until extubation. After midline laparotomy, the abdominal cavity was explored for signs of open perforation. Procedures beyond were dependent on intraoperative findings and are described in detail in the "Results" section.

### Outcome measures and statistical analysis

Therapeutic approaches, occurrence and severity of complications within 30 days after diagnosis, failure of initial therapy requiring alternative therapy or surgical revision, length of in-hospital stay, number of readmissions and requirement for parenteral nutrition were investigated. Data collection and analysis were performed with Microsoft Excel 2019, Microsoft Corporation, Redmond, Washington, USA and illustrated with GraphPad PRISM 9, Graphpad Software, Inc., San Diego, California, USA. Results in the manuscript and figures are reported as mean ± standard deviation (SD) with complete range.

## Results

### Patient characteristics, screening and study enrolment

**Figure 1:**
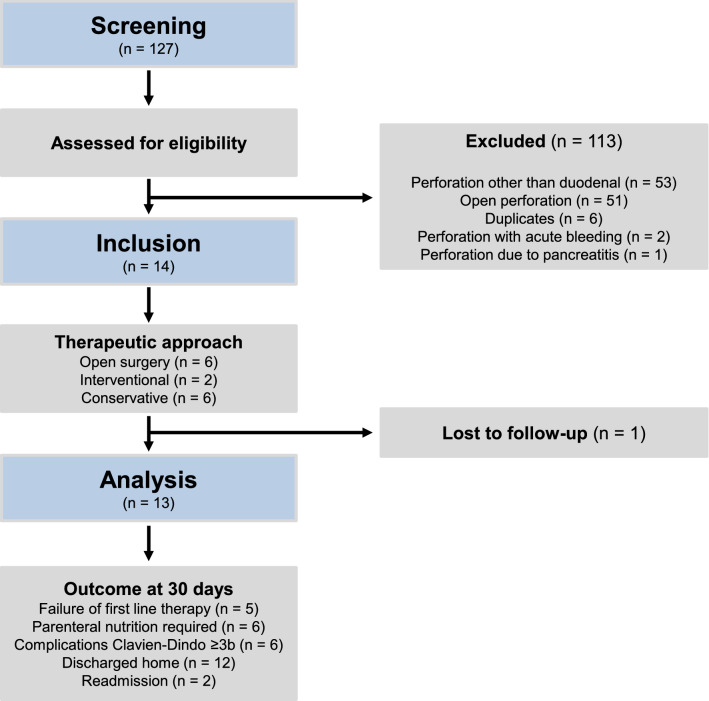
Screening, therapy decision, follow-up and analysis of patients. *n* number.

Details of patient screening and analysis are shown in Fig. 1. We identified 127 patients in total that had been treated for the ICD-10 diagnoses K26.0–K26.9 (duodenal ulcer) and K57.02–K57.93 (diverticulitis of the small intestine) at our institution between January 2010 and March 2021. One hundred thirteen patients were excluded from further analysis due to perforations of structures other than the duodenum (*n* = 53), open perforation into the abdominal cavity with peritonitis requiring emergency surgery (*n* = 51), duplicates (*n* = 6), perforation with acute bleeding requiring emergency surgery (*n* = 2), duodenal perforation diagnosed during open exploration for acute pancreatitis (*n* = 1). Fourteen patients fulfilled the inclusion criteria. Six patients were treated with open surgical therapy (42.9%), six patients received conservative therapy (42.9%) and two interventional treatment using endoscopic negative pressure therapy (14.3%). One patient being treated conservatively was referred to an external hospital shortly after initiation of therapy (7.1%) and therefore no follow-up data were available.

Detailed characteristics of treatment groups, patient-level comorbidities and medication are found in Table 1. Of the thirteen patients included in the final analysis ten were women (76.9%) and three men (23.1%). Mean age of the total patient cohort was 65 years. Patients that underwent surgery were between 29 and 75 years (mean 57.3 years, range 29–75 years) and therefore younger than the group of patients treated conservatively (mean 70.2 years, range 55—88 years) or interventionally (mean 75.5 years, range 69–82 years) respectively.

**Table 1:** Patient characteristics with relevant comorbidities and medication. *ASA:* acetylsalicylic acid; *BMI:* body mass index.

	Sex	Age	Comorbidities	Medication
**Surgical treatment (patients #1—#6)**				
#1	F	65	Enterothorax, arterial hypertension, phyllodes tumor of the breast, mastitis non-puerperalis	Candesartan
#2	F	66	Urinary tract infection, eosinophilic gastritis, hypothyroidism	Prednisolone, pantoprazole, L-thyroxine
#3	M	50	Mantle cell lymphoma	Aciclovir, cotrimoxazole, pantoprazole
#4	F	59	Type 2 diabetes mellitus, peripheral artery disease	Sitagliptin and metformin hydrochloride, lisinopril, ASA
#5	M	29	None	None
#6	F	75	Arterial hypertension	Bisoprolol, ramipril
**Interventional treatment (patients #7–#8)**				
#7	F	82	Atrial fibrillation, breast cancer	Apixaban
#8	F	69	Hypothyroidism, soft tissue rheumatic disorder	Dexamethasone
**Conservative treatment (patients #9—#13)**				
#9	F	75	Portal vein thrombosis, helicobacter pylori-gastritis, arterial hypertension, hypercholesterolemia	ASA, hydrochlorothiazide, candesartan, amlodipine, metoprolol, ezetimibe
#10	F	71	Hypercholesterolemia, coronary artery disease	ASA, nebivolol, venlaxfaxine, gabapentin
#11	F	62	Cachexia (BMI 13.6 kg/m^2^), hypercholesterinemia	ASA, simvastatin
#12	M	88	Prostate cancer, gastric ulcer, type 2 diabetes mellitus, renal insufficiency	sitagliptin, triamterene, metoprolol
#13	F	55	Chronic pain syndrome	None

### Examination results and assignment to therapy

Table 2 and Supplementary Data 1 show each patient’s case history and initial examination results including CT-imaging and, if performed findings during endoscopy, in detail. Seven patients (53.8%) reported a short history of acute abdominal or epigastric pain that had prevailed for less than 24 h prior to presentation at our emergency department. Symptoms had occurred three, four or ten days prior to admission in one patient each (7.7%). Two patients (15.4%) complained of abdominal or epigastric pain for four to five weeks before consulting a physician. Duration of symptoms in one patient (7.7%) suffering from aphagia, gastroesophageal reflux and singultus was not documented. Assessment and documentation of vital signs was performed in all patients showing mostly discrete abnormalities. One patient (7.7%) presented with arterial hypertension, another patient (7.7%) had febrile temperatures up to 38.7° C. Laboratory results showed elevated leukocytes in nine patients (69.2%) and conspicuous C-reactive protein (CRP) values in all but two patients (84.6%). There were no significant differences in leukocytes or CRP values when comparing results from patients treated conservatively, interventionally and surgically respectively.

**Table 2:** Findings of physical examination, laboratory assessment, computed tomography and endoscopy at initial presentation. *n/a:* not available*; bpm:* beats per minute*, CRP:* C-reactive protein, *CT* computed tomography.

	History	°C	Blood pressure mmHg	bpm	Leukocytes /µl	CRP mg/dl	CT	Endoscopy
**Surgical therapy in patients #1–#6**								
#1	Abdominal pain < 24 h	37.0	125/85	90	14,000	32.1	Gastric and colonic herniation to the thorax, covered perforation of the duodenum with retroperitoneal abscess, concomitant cholecystitis	Perforated diverticulum of pars descendens duodeni
#2	Epigastric pain for 10 days	36.6	130/80	84	11,580	14.7	Retroperitoneal perforation of duodenal ulcer	Not performed
#3	Aphagia, singultus, gastroesophageal reflux	36.6	115/60	88	4,820	5.2	Covered perforation of duodenal ulcer	Ulcus duodeni
#4	Abdominal and back pain < 24 h	37.3	130/85	76	18,950	44.4	Covered perforation of duodenal diverticulum	Not performed
#5	Epigastric pain for 5 weeks	36.0	100/60	96	15,060	23.2	Covered perforated duodenal ulcer	Not performed
#6	Pain, vomiting < 24 h	36.5	190/70	84	10,420	1.2	Perforated duodenal diverticulum with retroperitoneal abscess	Not performed
**Interventional therapy in patients #7–#8**								
#7	Epigastric pain, nausea < 24 h	36.6	182/97	82	6,300	0.3	Covered perforation of duodenal diverticulum	Perforated diverticulum of the duodenum
#8	Abdominal pain and vomiting for 3 days	36.2	123/83	77	14,200	33.9	Perforation of duodenal diverticulum	Perforated juxtapapillary diverticulum of the duodenum
**Conservative therapy in patients #9–#13**								
#9	Belt-like abdominal pain for 4 days	37.2	140/80	96	14,110	44.6	Covered perforation of duodenal pseudodiverticulum with concomitant partial thrombosis of the portal vein	Bile duct fistula to the duodenum suspected
#10	Abdominal pain < 24 h	n/a	n/a	82	6,000	42.0	Covered perforation of the duodenum	Not performed
#11	Abdominal pain for 4 weeks	37.0	150/70	92	16,420	0.3	Covered perforation of duodenal ulcer	Ulcus duodeni
#12	Abdominal pain < 24 h	38.7	145/85	60	9,900	2.2	Perforation of duodenal diverticulum	Not performed
#13	Epigastric pain < 24 h	37.3	113/81	74	15,270	5.1	Perforated duodenal ulcer with retroperitoneal abscess	Ulcus duodeni

**Figure 2:**
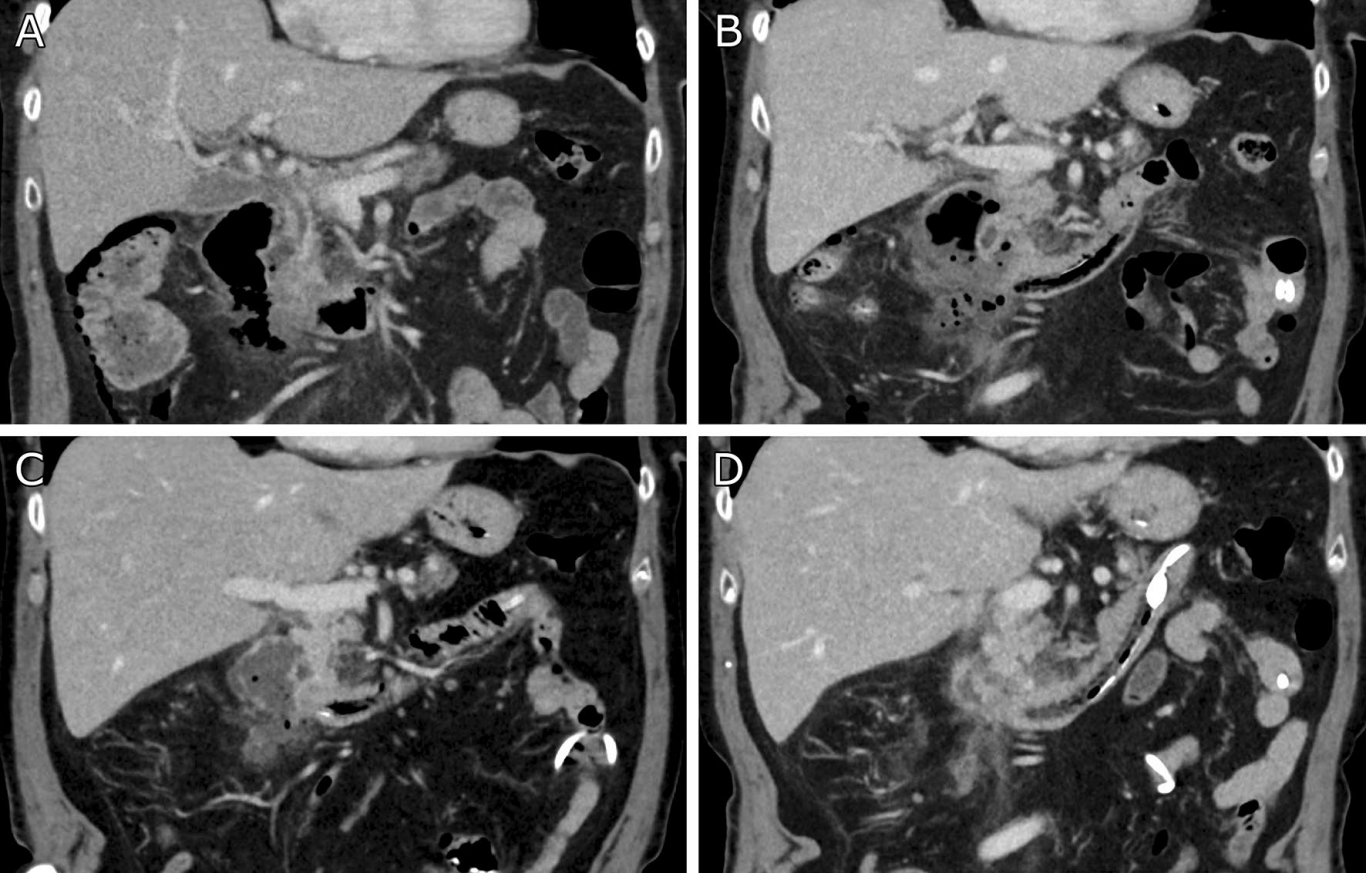
Time course of regression of a retroperitoneal abscess due to perforation of a duodenal diverticulum in **patient #8** at initial diagnosis with retroperitoneal gas collections (**A**), with incipient size decrease and regressing gas collections after four days (**B**), further size reduction and decreasing perifocal inflammation after nine days (**C**), and increasingly consolidated state after 15 days (**D**) with endoscopic negative pressure therapy.

Six (46.2%) patients were treated surgically for contained perforations of a duodenal diverticulum or duodenal ulcer. Surgery was performed on the day of diagnosis in one (7.7%) patient, whereas two (15.4%) patients were operated on the first day and three (23.1%) patients on the second day following diagnosis. Antibiotic therapy in patients that were treated without surgery was initiated on the day of diagnosis in four (30.8%) patients, with a 24-h delay in two (15.4%) patients and started two days prior to diagnosis as calculated therapy in one patient (7.7%). Endoscopic negative pressure therapy was initiated the day of presentation in one patient (7.7%) and one day after admission in the second patient respectively. Figure 2 exemplary presents the course of an interventionally treated patient shown by consecutive CT imaging.

Table 3 shows a brief overview of treatment parameters, length of stay, adverse events and frequency of readmissions according to therapy. Detailed information on each patient is given in Supplementary Document 1.

**Table 3:** Description of therapy and treatment failure, antibiotic therapy, microbiological findings, length of stay, adverse epvents according to Clavien-Dindo classification and readmissions.

	First therapy	Second therapy	Antibiotic therapy	Microbiology	Parenteral nutrition	Length of stay	Adverse events (frequency)	Readmission within 30 days
**Surgical therapy in patients #1–#6**								
#1	Abscess evacuation, sewing of duodenal perforation, cholecystectomy, repositioning of herniated stomach and colon, hiatoplasty; endoscopic negative pressure therapy	Resection of insufficient duodenal segment, drainage by attachment of duodenojejunostomy	Meropenem vancomycin fluconazole	Veillonella parvula and dispar Escherichia coli, Streptococcus anginosus, Proteus mirabilis	Yes	22 days	IIIb (1)	No
#2	Billroth II gastrectomy	Intestinal feeding with negative pressure therapy at duodenal stump; Open abdominal lavage endoscopic negative wound pressure therapy	ciprofloxacin, metronidazolemeropenem vancomycin fluconazole	Escherichia coli, Enterococcus faecium and faecalis, Klebsiella pneumoniae, Streptococcus mitis and anginosus, Prevotella buccae, Staphylococcus haemolyticus, Leuconostoc species, Lactobacillus rhamnosus and paracasei	Yes	22 days	II (1) IIIa (1) IVa (1)	No
#3	Billroth II gastrectomy	No	ampicillin/sulbactam fluconazole meropenem vancomycin anidulafungin cotrimoxazole	Candida glabrata Enterococcus faecium and Citrobacter freundii	Yes	12 days	IVa (1)	No
#4	Diverticulum resection, cholecystectomy, insertion of Kehr’s tube into the biliary duct and sewing of the duodenum	Pancreatectomy, splenectomy and cholecystectomy	meropenem, vancomycin, fluconazole linezolid fluconazole	Candida albicans Enterococcus faecium	No	19 days	IVa (1)	No
#5	Open abscess evacuation and drainage	Endoscopic transgastric drainage	piperacillin/tazobactam	Streptococcus constellatus Mycobacterium tuberculosis complex	No	10 days	IIIb (1)	Yes
#6	diverticulum resection, cholecystectomy, insertion of Kehr’s tube into the biliary duct and sewing of the duodenum	No	piperacillin/tazobactam	Proteus mirabilis, Klebsiella pneumonia, Lactobacillus species, Bacteroides ovatus	No	15 days	No	No
**Interventional therapy in patients #7–#8**								
#7	Endoscopic negative pressure therapy	No	piperacillin/tazobactam fluconazole	Enterococcus faecium	Yes	20 days	I (1)	No
#8	Endoscopic negative pressure therapy	No	Cefotaxime metronidazole fluconazole	None	Yes	20 days	No	No
**Conservative therapy in patients #9–#13**								
#9	Conservative	No	piperacillin/tazobactam fluconazole	Helicobacter pylori	No	12 days	No	No
#10	Conservative	No	meropenem vancomycin fluconazole	None	No	12 days	No	No
#11	Conservative	No	piperacillin/tazobactam fluconazole amoxicillin, clarithromycin	Helicobacter pylori	No	11 days	No	No
#12	Conservative	No	ciprofloxacin metronidazole	None	No	10 days	No	No
#13	Conservative	ERCP	ciprofloxacin metronidazole piperacillin/tazobactam	None	No	9 days 14 days	IIIb (1)	Yes

### Length of stay, complications and readmissions

**Figure 3:**
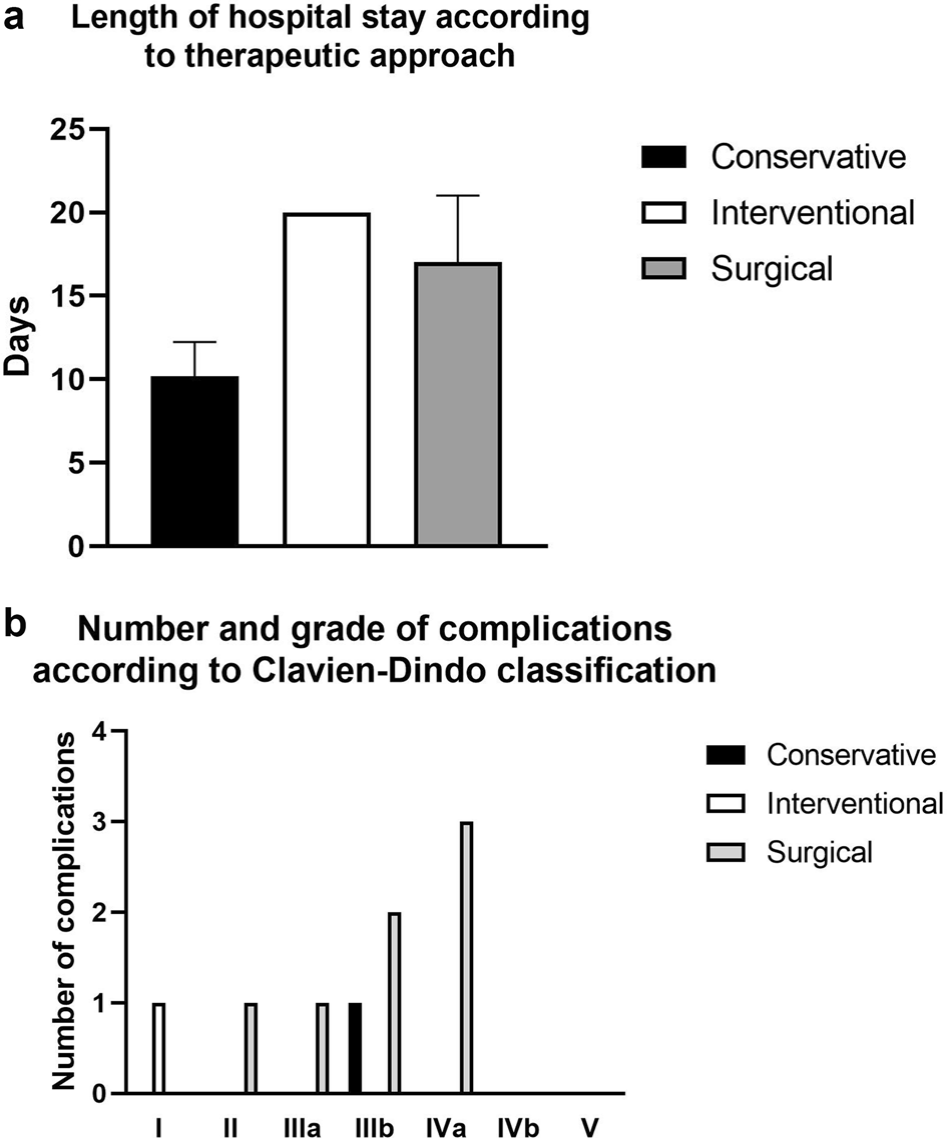
(**a**) Length of stay in days according to type of therapy. (**b**) Distribution and number of adverse events according to Clavien-Dindo classification of postoperative complications.

Length of hospital stay disregarding length of stay after readmission was between nine and 22 days. Mean length of stay was 10.2 days for conservative therapy, 20.0 days for interventional therapy and 17 days for surgical therapy. Further information is given in Fig. 3a.

None of the patients treated conservatively or interventionally was admitted to the intensive care unit, whereas three (50.0%) patients that underwent surgery required intensive care. There were two readmissions for further inpatient treatment, one following surgical and conservative treatment each, whereas no readmission occurred after interventional treatment. The extent of secondary hospital stay was 29 days for **patient #5,** who underwent endoscopic transgastric drainage for persistent retrogastric abscess and further antibiotic treatment for infection with Mycobacterium tuberculosis following open abscess evacuation and drain insertion. **Patient #13** underwent endoscopic drainage of a persistent retroperitoneal abscess and had fourteen days of rehospitalization after initial conservative treatment.

Distribution of complications according to Clavien-Dindo classification of postoperative complications is shown in Fig. 3b. In total, there were nine complications, which were grade I, II and IIIa each, three complications graded IIIb and three complications graded IVa in seven patients, five of whom (83.3%) underwent surgical therapy and two (28.6%) non-surgical treatment. **Patient #1**, that underwent open duodenal sewing of the perforated segment, cholecystectomy, repositioning of the herniated stomach and colon and hiatoplasty, had revision laparotomy 4 days after initial surgery which showed insufficiency of the duodenal sewing (grade IIIb according to Clavien-Dindo classification of postoperative complications). The insufficient duodenal segment was partially resected and drained by attachment of a duodenojejunostomy. After a total length of stay of 22 days the patient was discharged home without readmission or occurrence of further complications. **Patient #2** experienced three complications that were graded II, IIIa and IVa respectively according to Clavien-Dindo classification of postoperative complications^22^ following Billroth’s operation II. Due to biliary leakage and postoperative paralytic ileus a tube for intestinal feeding in combination with a tube placed at the duodenal stump for negative pressure therapy was inserted by endoscopy three days after surgery (grade IIIa). The fourth postoperative day, deterioration of the patient’s general state as well as increasing leukocyte count and CRP led to CT-scan depicting duodenal stump insufficiency with local peritonitis as well as lung artery embolism (grade II). Open surgical exploration was indicated revealing ongoing duodenal leakage which was treated by abdominal lavage and endoscopic placement of a tube for negative wound pressure. Following surgery, the patient was admitted to intensive care unit (grade IVa) for therapeutic anticoagulation and broad-spectrum antibiotic therapy. After 22 days of steady recovery the patient was discharged. **Patient #3** underwent exploratory laparotomy with Billroth’s operation II 5 days following diagnosis. Respiratory insufficiency and pulmonal sepsis based on *Pneumocystis jirovecii*-pneumonia and anemia 4 days following resection necessitated transfer of the patient to intensive care unit, transfusion of blood and expansion of antimicrobial therapy by cotrimoxazole (grade IVa). After 12 days the patient was admitted to the ear, nose and throat department for treatment of sinusitis. In **patient #4**, diverticulum resection, cholecystectomy, insertion of Kehr’s tube into the biliary duct and sewing of the duodenum was performed. Biliary leakage urged revision laparotomy, pancreatectomy, splenectomy and cholecystectomy, which was performed two days following initial surgery, and postoperative treatment at intensive care unit (grade IVa). Nineteen days after admission and initial surgical treatment the patient was discharged home. **Patient #5** was treated by laparotomy and open abscess evacuation with drainage placement. Due to retrogastric abscess formation 9 days later, an endoscopic transgastral drain was inserted (grade IIIb). Due to identification of *Mycobacterium tuberculosis* from abscess content, the patient was admitted to the department of Internal Medicine after 10 days for further treatment.

In **patient #7** negative wound pressure therapy was performed. Following 5 endoscopies over a period of 15 days and antibiotic therapy for urinary tract infection (grade I), the patient was discharged after 20 days length of total stay. **Patient #13** was treated conservatively due to perforated duodenal ulcer with retroperitoneal abscess. Eleven days later, CT scan showed constant abscess formation. Therefore, the patient received underwent endoscopy retrograde cholangiopancreatography with internal drainage of the abscess (grade IIIb). The patient was discharged after 14 days.

## Discussion

To our knowledge, this is the to date most comprehensive retrospective cohort study of contained duodenal perforations and their treatment. The study comprises thirteen patients with covered duodenal perforation to the retroperitoneum and reports on conservative, interventional or surgical treatment respectively. Length of stay was shorter in patients that were treated conservatively as compared to patients that underwent surgical or interventional therapy. Of note, there was no failure of primary therapy in the group of patients treated conservatively or interventionally, whereas four (66.7%) of six patients required revision surgery following primary surgical intervention. Additionally, conservative and interventional treatment were associated with fewer complications (one grade I and IIIb according to Clavien-Dindo classification each) in contrast to surgical therapy (total seven adverse events graded II (one patient), IIIa (one patient), IIIb (two patients) and IVa (three patients)).

Despite considerable morbidity and mortality^4,7^ entailed by surgery for duodenal perforation to the retroperitoneum, this had been the most frequent therapy as found in the literature^4–10^. The extent of surgery and required technique depend on the size and location of the perforation and comprise several approaches including primary closure with or without omental flap, creation of gastrojejunostomy to bypass the duodenum or reconstructive surgery to create a duodenoduodenostomy, Roux-Y-duodenojejunostomy, Billroth II gastrectomy or (partial) duodenocephalopancreatectomy^23–25^.

In contrast, evidence from conservative treatment in patients with perforated peptic ulcers shows a success rate for non-operative therapy of 50% – 70%^11,12^. Recent case reports^13,14^ and patient series report similar findings: Rossetti et al. performed a retrospective analysis^7^ akin to the one presented by us, reporting on seven patients with contained perforation of the duodenum. Of these patients six (85.7%) underwent open surgery and one (14.3%) patient received a nasogastric tube and antibiotic therapy. Conservative therapy was chosen due to mild symptoms and entailed a favorable clinical course with 15 days of hospitalization. Of the surgical patients, one (14.3%) died and one developed a biliary leak following successful excision and suture of a duodenal perforation. The remaining five patients had a mean hospital stay of 22.4 days.

Conservative treatment is however poorly defined and the significance each of the components has is unknown. Whether there is value in cessation of oral intake and temporary parenteral nutrition and for what duration remains to be answered and cannot be decided with the available data, but should be addressed in future investigations. Empiric antibiotic therapy is mostly of broad-spectrum covering bacteria from the upper gastrointestinal tract but omitting fungi. More narrow antibiotic therapies or de-escalation adapted to microbial findings has not been reported. In our cohort, most bacterial cultures were polymicrobial. With regard to our culture results we recommend empiric broad-spectrum antibiotics with coverage of both gram-positive and -negative bacteria, anaerobes and candida species. In case helicobacter pylori is detected, eradication therapy is warranted and should be tapered to local resistance patterns^26,27^. In one patient readmitted due to recurrent retroperitoneal abscess formation, Mycobacterium tuberculosis was unexpectedly discovered, requiring tuberculostatic therapy. In the event of extraordinary and recurrent disease, gastrointestinal tuberculosis should be kept in mind as a potential cause of perforation and appropriate diagnostics and therapy initiated.

In the current literature, there is evidence supporting endoscopic negative pressure therapy for various defects of hollow visceral organs^16,28–30^, also suggesting that its implementation in the therapy of duodenal perforations could be beneficial. Over the recent years endoscopic treatment of gastrointestinal perforations has evolved and heavily influenced the therapeutic strategy for iatrogenic perforations^16,29^, oesophageal leakage^31^ and defects of the upper gastrointestinal system in general^18,20,28,30,32^. Depending on the location and size of wall discontinuity, clip application, stent implantation, endoscopic suturing or negative wound pressure therapy have been applied^18,20,29,30^. Endoscopic vacuum therapy for leaks of the upper gastrointestinal system has demonstrated promising results with success rates of 70% – 100%^19,32^. Despite the 2010 guideline of the *American Society of Gastrointestinal Society* advising against endoscopic treatment of perforated duodenal ulcers^26^, additional endoscopic negative pressure therapy for the conservative treatment of self-contained perforations of the duodenum has been suggested as a therapeutic alternative. Our cohort includes two patients with spontaneous perforation of the duodenum that were successfully treated by endoscopic negative pressure therapy as stand-alone treatment, which we have already published in a separate case report^21^. Loske et al. recently reported about their successful treatment of iatrogenic duodenal perforations and insufficient sutures as well as anastomoses of the duodenum by endoscopic negative pressure therapy^18,20,33^. Likewise, in our cohort **patient #2**, who experienced duodenal stump leakage following Billroth’s operation II for retroperitoneal perforation of duodenal ulcer, was successfully treated by endoscopic negative pressure therapy in addition to open abdominal lavage. However, if results from endoscopic negative pressure therapy of iatrogenic duodenal perforations are readily transferrable to the treatment of spontaneous perforations is not proven so far.

Of note, our analysis comes with relevant limitations due to the retrospective study design and the small and heterogeneous set of patients. Further, allocation bias due to missing predefined selection criteria for each therapeutic approach cannot be excluded, and treatment allocation was based on subjective patient assessment by the treating surgeon only. Patients treated without surgery were older and patients with more severe symptoms might have been preferentially treated with surgery. On the other hand, conservative and interventional treatment without therapy failure may have been related to a less severe course of disease and not necessarily connected to more effective therapy. Whether patients treated by endoscopic negative pressure therapy might have recovered similarly with conservative treatment cannot be excluded. Although both conservative and interventional treatment appear feasible and even advantageous in a subset of patients, criteria for therapy stratification remain undetermined to date. Early diagnosis, stable vital signs without signs of worsening sepsis and manageable abdominal pain have been recommended as crucial factors^13,14,34,35^. So far, there are no prospective clinical trials assessing conservative, interventional and surgical therapy for contained duodenal perforation in a randomized patient cohort. Given the relatively low frequency of duodenal perforations to the retroperitoneum and limited case numbers, only small retrospective cohort studies would be possible. Nevertheless, designing an appropriate prospective clinical trial to provide robust evidence should be considered.

In conclusion, surgery for confined perforations of the duodenum mostly requires high-risk interventions, incurring morbidity, reoperations and poor quality of life. To date, available evidence from case reports and retrospective case series endorses a non-surgical approach in selected patients diagnosed with duodenal perforation into the retroperitoneum. The significance of endoscopic negative pressure therapy is emerging so far, but it stands to reason that it most likely constitutes an appropriate therapy option considering the benefits from treatment of anastomotic leakages and perforations of the gastrointestinal tract in general.

## Supplementary Information

Supplementary Information.

## Data Availability

The data analyzed during the presented study are available on request from the corresponding author.

## Abbreviations

ASA: Acetylsalicylic acid
BMI: Body mass index
bpm: Beats per minute
CRP: C-reactive protein
CT: Computed tomography
ICD: International classification of disease
