# Supplementary material for: Retrospective analysis of different therapeutic approaches for retroperitoneal duodenal perforations
Source: Sci Rep. 2022 Jun 17;12:10243. doi: 10.1038/s41598-022-14278-8 (PMC9205956; doi:10.1038/s41598-022-14278-8)
Supplement: Supplementary file 1 — Supplementary Information. [file 41598_2022_14278_MOESM1_ESM.docx]

## ***Surgical therapy***

**Patient #1** was admitted to the emergency department due to a short history of acute epigastric pain and dyspnea. CT scan revealed gastric and colonic herniation to the thorax as well as covered perforation of the duodenum with retroperitoneal abscess and concomitant cholecystitis. Persistent pain despite analgesia caused open surgical exploration to be indicated. CT scan findings were confirmed so that evacuation of retroperitoneal abscess, sewing of the duodenal perforation, cholecystectomy, repositioning of the herniated stomach and colon and hiatoplasty were performed. During surgery an intraluminal tube for negative pressure therapy was placed under endoscopic control. A persistent biliary leak caused negative wound pressure therapy to be terminated and revision laparotomy was performed four days later, which showed insufficiency of the duodenal sewing (Grade IIIb according to Clavien-Dindo classification of postoperative complications). The insufficient duodenal segment was partially resected and drained by attachment of a duodenojejunostomy. Treatment was accompanied throughout by broad-spectrum antibiotic therapy with meropenem, vancomycin and fluconazole and parenteral nutrition. Microbiology sampling detected *Veillonella parvula* and *dispar*, *Escherichia coli*, *Streptococcus anginosus* and *Proteus mirabilis*. After a total length of stay of 22 days the patient was discharged home without readmission or occurrence of further complications.

Following a history of abdominal pain for ten days, **patient #2** was diagnosed with retroperitoneal perforation of a duodenal ulcer, so that an open Billroth’s operation II was performed and subsequent calculated antibiotic therapy with ciprofloxacin and metronidazole and parenteral nutrition initiated. Due to biliary leakage and postoperative paralytic ileus a tube for intestinal feeding in combination with a tube placed at the duodenal stump for negative pressure therapy was inserted by endoscopy three days after surgery (Grade IIIa). On the fourth postoperative day, deterioration of the patient’s general state as well as an increasing leukocyte count and CRP led to a CT scan depicting duodenal stump insufficiency with local peritonitis as well as lung artery embolism (Grade II). Open surgical exploration was indicated and revealed ongoing duodenal leakage, which was treated by abdominal lavage and endoscopic placement of a tube for negative wound pressure. Following surgery, the patient was admitted to the intensive care unit (Grade IVa) for therapeutic anticoagulation and broad-spectrum antibiotic therapy with meropenem, vancomycin and fluconazole. *Escherichia coli, Enterococcus faecium and faecalis, Klebsiella pneumoniae, Streptococcus mitis* and *anginosus, Prevotella buccae, Staphylococcus haemolyticus, Leuconostoc species, Lactobacillus rhamnosus* and *paracasei* were detected. After 22 days of steady recovery the patient was discharged.

In the course of evaluation for allogeneic bone marrow transplantation due to recurrent mantle cell lymphoma, **patient #3** reported aphagia, gastroesophageal reflux and singultus for an unknown period of time and was diagnosed with sinusitis maxillaris, aspiration pneumonia and covered perforation of a duodenal ulcer. After initiation of calculated antibiotic therapy with ampicillin/sulbactam and fluconazole and surgical consultation, exploratory laparotomy with Billroth’s operation II was performed five days following diagnosis. Parenteral nutrition was temporarily administered and antibiotic therapy was escalated to meropenem, vancomycin and anidulafungin because of microbiological detection of *Candida glabrata, Enterococcus faecium* and *Citrobacter freundii*. Respiratory insufficiency and pulmonary sepsis based on *Pneumocystis jirovecii*-pneumonia and anemia four days following resection necessitated transferring the patient to the intensive care unit, blood transfusion and expansion of antimicrobial therapy by cotrimoxazole and substitute (Grade IVa). After 12 days the patient was admitted to the Ear, Nose and Throat Department for further treatment of sinusitis.

**Patient #4** was diagnosed with a covered perforation of duodenal diverticulum at an external hospital after a short history of abdominal pain and was admitted to our hospital for antibiotic therapy with meropenem, vancomycin and fluconazole and diverticulum resection, cholecystectomy, insertion of Kehr’s tube into the biliary duct and sewing of the duodenum. Biliary leakage prompted revision laparotomy, pancreatectomy, splenectomy and cholecystectomy, which were performed two days following initial surgery, and postoperative treatment at the intensive care unit (Grade IVa). Detection of *Candida albicans* and *Enterococcus faecium* allowed de-escalation to linezolid and fluconazole. Nineteen days after admission and initial surgical treatment the patient was discharged home.

**Patient #5** presented at an external hospital with a five-week history of epigastric pain that was caused by subhepatic abscess formation originating from a perforated duodenal ulcer. The patient was transferred to our hospital, where open abscess evacuation and drainage was performed. Following calculated antibiotic therapy with piperacillin/tazobactam and detection of *Streptococcus constellatus* only, the patient was discharged after ten days of hospital stay. Nine days later, the patient presented at an external hospital again due to new epigastric pain caused by retrogastric abscess formation. After being transferred to our hospital, an endoscopic transgastral drain was inserted (Grade IIIb). Following identification of *Mycobacterium tuberculosis* from abscess content, the patient was admitted to the Department of Internal Medicine after ten days for further treatment.

Abdominal pain for several hours and vomiting brought patient **#6** to our emergency department, where a perforated duodenal diverticulum with retroperitoneal abscess was diagnosed. Exploratory laparotomy with diverticulum resection, cholecystectomy, insertion of Kehr’s tube into the biliary duct and sewing of the duodenum were performed. Antibiotic therapy with piperacillin/tazobactam was initiated in view of results from microbiological sampling yielding *Proteus mirabilis, Klebsiella pneumonia, Lactobacillus species and Bacteroides ovatus*. After 15 days of hospitalization the patient was discharged home with an inserted Kehr’s tube, which was removed 34 days later.

## ***Interventional therapy***

**Patient #7** was brought to our emergency department with acute postprandial abdominal pain. After CT scan with signs of covered perforation of a duodenal diverticulum, calculated antibiotic therapy with piperacillin/tazobactam and fluconazole as well as parenteral nutrition was commenced and endoscopy was performed to place a drain in order to introduce negative wound pressure therapy. Following five endoscopies over a period of 15 days and antibiotic therapy for urinary tract infection, the patient was discharged after a total of 22 days hospitalization.

**Patient #8** presented to an external hospital with a history of epigastric pain and vomiting for two days. CT imaging showed perforation of duodenal diverticulum. After referral to our hospital, we initiated parenteral nutrition and empiric antibiotic therapy with cefotaxim, metronidazole and fluconazole and performed endoscopic negative pressure therapy for 14 days. Discharge was possible after 20 days of hospitalization. Figure 2 shows the CT findings at admission and after 4, 9 and 15 days.

## ***Conservative therapy***

**Patient #9** was admitted to the emergency department with first time occurrence of postprandial belt-like epigastric pain. CT scan revealed covered perforation of duodenal pseudodiverticulum with concomitant partial thrombosis of the portal vein. We therefore introduced therapeutic anticoagulation and calculated antibiotic therapy with piperacillin/tazobactam and fluconazole. After 12 days, the patient was discharged home with a recommendation for Helicobacter pylori eradication therapy based on microbiological diagnosis.

**Patient #10** was diagnosed with covered perforation of the duodenum by ambulant CT imaging for clarification of a short history of epigastric pain and presented to our emergency department for further treatment. We introduced antibiotic therapy with meropenem, vancomycin and fluconazole. Following 12 days of hospitalization the patient was discharged home.

**Patient #11** was suspected of having a peritoneal metastasized tumor originating from the duodenum because of initial presentation with intestinal obstruction, excessive weight loss and kachexia with a BMI of 13.6 kg/m² following four weeks of epigastric pain. Diagnostic workup revealed covered perforation of a duodenal ulcer caused by Helicobacter pylori. Malignancy was ruled out by biopsy. We initially introduced antibiotic therapy with piperacillin/tazobactam and fluconazole and shifted to amoxicillin and clarithromycin for eradication therapy and discharged the patient after 11 days.

**Patient #12** presented to the emergency service with a short history of epigastric pain with fever. Perforation of a duodenal diverticulum caused us to introduce antibiotic therapy with ciprofloxacin and metronidazole. The patient was discharged after ten days of conservative treatment.

**Patient #13** was transferred to our hospital because of ex domo diagnosis of a perforated duodenal ulcer with retroperitoneal abscess. Antibiotic therapy with ciprofloxacin and metronidazole supported rapid recovery so that the patient was discharged home after nine days. Two days later, epigastric pain had occurred anew. CT scan showed constant abscess formation. The patient therefore received antibiotic therapy with piperacillin/tazobactam and an endoscopy retrograde cholangiopancreatography was performed with internal drainage of the abscess IIIb. The patient was discharged after 14 days.
